# Urinary Cadmium as a Marker of Exposure in Epidemiological Studies

**DOI:** 10.1289/ehp.1307376

**Published:** 2013-10-01

**Authors:** Scott V. Adams, Polly A. Newcomb

**Affiliations:** 1 Cancer Prevention Program, Public Health Sciences Division, Fred Hutchinson Cancer Research Center, Seattle, Washington, USA; 2 Department of Epidemiology, School of Public Health, University of Washington, Seattle, Washington, USA

Urinary cadmium (U-Cd) is commonly interpreted in epidemiological studies to measure cadmium accumulated in the kidney, and is thus used as a marker of long-term exposure. This concept is based primarily on occupational cohorts exposed to high Cd levels, and its generalization to populations chronically exposed to lower environmental Cd levels—primarily through tobacco and foods grown on contaminated soil—is of central importance to studies of health outcomes, including heart disease, cancer, kidney disease, and osteoporosis, that have been associated with Cd (Järup and Åkesson 2009). In their article, Chaumont et al. (2013) described complications with understanding Cd body burden from U-Cd. However, several items in the article would benefit from clarification.

Evidence presented by Chaumont et al. (2013) included stratified plots of log-transformed U-Cd with age, comparing men and women and, separately, by smoking status. The authors observed an approximately constant offset between current smokers and nonsmokers, which they interpreted to mean that the difference in U-Cd did not change with age—in contrast to the expectation based on U-Cd reflecting accumulation of Cd in the kidney. However, a constant offset between curves on a log-scale implies that the ratio, not the difference, is constant. Because the curves have generally upward trends, the difference in U-Cd between current smokers and nonsmokers must be increasing.

Furthermore, Figure 2 and Table 2 of Chaumont et al. (2013) showed higher mean U-Cd in former smokers compared with never-smokers over a range of ages, as expected if U-Cd reflects, at least in part, cumulative exposure. Nonetheless the authors stated, “We observed no differences … between former and never-smoker adults.” The difference did not reach statistical significance, which might be explained by the modest sample size and broad distribution of U-Cd in former smokers, who were more often male than never-smokers and who may have varied widely in smoking dose and may have quit smoking years before the study.

A more informative investigation of the association of U-Cd with smoking would use multivariate regression, controlling for these variables. Many studies from a variety of populations have demonstrated higher U-Cd in former smokers (Adams and Newcomb 2013; Adams et al. 2011; Gunier et al. 2013; McElroy et al. 2007; Olsson et al. 2002; Paschal et al. 2000).

Chaumont et al. (2013) concluded that estimates of dietary Cd intake from food contamination data might be more useful than U-Cd for exposure assessment. It seems unlikely that individual-level exposure measurement based on dietary recall would be superior to measurement of U-Cd whether U-Cd reflects long-term or recent exposure, or a combination. Chaumont et al. (2013) highlighted the importance of carefully considering human Cd physiology, particularly in children and adolescents. Clearly the relationship between U-Cd and Cd exposure is complex. Yet the results of Chaumont et al. do not warrant abandonment of U-Cd as a measure of environmental exposure for epidemiological studies.
